# Assessment of Offspring DNA Methylation across the Lifecourse Associated with Prenatal Maternal Smoking Using Bayesian Mixture Modelling

**DOI:** 10.3390/ijerph121114461

**Published:** 2015-11-13

**Authors:** Frank de Vocht, Andrew J Simpkin, Rebecca C. Richmond, Caroline Relton, Kate Tilling

**Affiliations:** 1 School of Social and Community Medicine, University of Bristol, Bristol BS8 2PS, UK; E-Mails: Andrew.Simpkin@bristol.ac.uk (A.J.S.); Rebecca.Richmond@bristol.ac.uk (R.C.R.); Caroline.Relton@bristol.ac.uk (C.R.); Kate.Tilling@bristol.ac.uk (K.T.); 2 MRC Integrative Epidemiology Unit, University of Bristol, Bristol, BS8 2BN, UK; 3 Institute of Genetic Medicine, Newcastle University, Newcastle upon Tyne NE1 3BZ, UK

**Keywords:** ALSPAC, Bayesian, DNA methylation, epigenetics, longitudinal data, mixture modelling, pregnancy, smoking

## Abstract

A growing body of research has implicated DNA methylation as a potential mediator of the effects of maternal smoking in pregnancy on offspring ill-health. Data were available from a UK birth cohort of children with DNA methylation measured at birth, age 7 and 17. One issue when analysing genome-wide DNA methylation data is the correlation of methylation levels between CpG sites, though this can be crudely bypassed using a data reduction method. In this manuscript we investigate the effect of sustained maternal smoking in pregnancy on longitudinal DNA methylation in their offspring using a Bayesian hierarchical mixture model. This model avoids the data reduction used in previous analyses. Four of the 28 previously identified, smoking related CpG sites were shown to have offspring methylation related to maternal smoking using this method, replicating findings in well-known smoking related genes *MYO1G* and *GFI1*. Further weak associations were found at the *AHRR* and *CYP1A1* loci. In conclusion, we have demonstrated the utility of the Bayesian mixture model method for investigation of longitudinal DNA methylation data and this method should be considered for use in whole genome applications.

## 1. Introduction

Epigenetics, the study of genome modifications not involving changes in the nucleotide sequence, offers the potential to identify molecular mechanisms by which environmental and lifestyle exposures may affect health [1,2]. Epigenetic mechanisms include DNA methylation, histone modifications and microRNA, all of which act in concert to regulate gene expression [3]. DNA methylation, the addition of methyl groups to nucleotide bases, is the most stable and most readily quantifiable epigenetic mark and has thus become the most widely studied. Recent technological advances have allowed the application of genomic technologies to epigenetics, facilitating the large scale generation of quantitative DNA methylation data across the genome [4]. 

Maternal smoking during pregnancy has been shown to expose the foetus to the harmful chemicals resulting from maternal use of tobacco through placental transfer and has been associated with reduced birthweight [5] and preterm birth [6]. Suboptimal growth has in turn been linked to increased risk of cardiovascular disease, diabetes mellitus type 2, dyslipidemia and end-stage renal disease in adulthood [7], which may adversely affect reproductive health of offspring [8], and may affect intelligence and cognitive development [9]. Despite these risks to newborns (as well as known risks of tobacco smoking for the mothers), in England about 12% of pregnant women are still smoking at the time of delivery [10], with similar prevalence reported in other western, high-income countries [11,12]. Cigarette smoke is known to be associated with DNA methylation [13,14,15], and since higher DNA methylation levels in the foetus have also been demonstrated in the genes involved in developmental processes [16,17] associated with maternal smoking during pregnancy, this seems to indicate a mediating role of epigenetic processes. Interestingly, data indicate that the effect of in utero exposure on foetus’ DNA methylation is stronger when the mother smoked past 18 weeks than when the mother stopped smoking earlier in pregnancy; perhaps when knowledge of the pregnancy occurred [18].

It has also been shown that early cessation of smoking can reverse (some of) the environmentally responsive influences on birthweight and lower the risk of preterm birth [19], and this aligns with data indicating that DNA methylation may be reversible. However, certain methylation patterns related to maternal smoking during pregnancy can still be observed in peripheral blood of offspring as children and adolescents [20,21] implying, for smoking, lasting effects. There has been relatively little work modelling methylation changes over time [22,23,24,25], but these lasting effects are still to be confirmed since at present it cannot be excluded that (some of) the observed effects could have been the result of postnatal exposure to environmental tobacco smoke from, for example, second hand smoke, or from adolescents smoking themselves [26,27,28].

A previous study, based on the Accessible Resource for Integrated Epigenomic Studies (ARIES) data [29] from the Avon Longitudinal Study of Parents and Children (ALSPAC) [30,31], and conducted by some of the authors involved in this work, evaluated the association between maternal persistent smoking during pregnancy and DNA methylation at birth, childhood and adolescence, taking into account postnatal effects of environmental tobacco smoke exposure [24]. The authors observed differential methylation at 15 CpG sites in seven gene regions (*AHRR*, *MYO1G*, *GFI1*, *CYP1A1*, *CNTNAP2*, *KLF13 and ATP9A*) at birth [24]. Of these, *GFI1*, *KLF13* and *ATP9A* showed reversibility of methylation at later time points, while *AHRR*, *MYO1G*, *CYP1A1* and *CNTNAP2* showed persistent perturbed patterns throughout childhood and adolescence. Six of these seven gene regions confirmed findings from other EWAS (Epigenome-Wide Association Studies) [16,17], and the top hit, cg05575921, was also previously associated with maternal smoking and methylation of DNA in both cord and neonatal blood [16]. Cg05575921 is located at the *AHRR* gene and has been widely reported as a smoking responsive DNA methylation locus [16,26]. 

An important limitation of this study was that even though only 28 CpG sites (of over 485,000) were identified as being differentially methylated, because of localized clustering of CpG sites in gene regions further selection of seven sites (one per gene) was made, and Richmond *et al.* focussed on the seven top CpG sites; one in each of seven identified gene regions: *AHRR* (cg05575921), *MYO1G* (cg22132788), *GFI1* (cg09935388), *CYP1A1* (cg05549655), *CNTNAP2* (cg25949550), *KFL13* (cg26146569), and *ATP9A* (cg07339236). 

Taking all differentially methylated sites forward would have resulted in convergence problems as a result of the collinearity within data, which (in general) will result in models not converging or, if they do to result in inflated estimates and standard errors [32]. The latter can be addressed by including some form of penalization to the models, and the BMM described here does this in a Bayesian context by *a priori* assuming that maternal smoking during pregnancy has no effect on a proportion of CpG sites. We explore whether in the context of such highly correlated, cross-sectional, data that required two dimensionality reduction steps, a Bayesian mixture model (BMM) previously developed for the analyses of gene-environment interactions [32], and subsequently also evaluated in the context of highly correlated environmental exposure mixtures [33], may be beneficial. More specifically, we evaluate whether the use of the BMM results in improved inferences by not including the second dimensionality reduction step; thereby opening up the possibility that the original single site analyses may have resulted in false positive findings, because of high correlation with a causal CpG site, or in missed hits as a result of a collapse of information [34]. 

## 2. Experimental Section

### 2.1. Data

These analyses are based on the dataset used by Richmond *et al.* [24], and include genome-wide DNA methylation data collected from children at birth (cord blood) and at ages 7 and 17 (whole blood) available from the Accessible Resource for Integrated Epigenomic Studies (ARIES) [29]. This study is nested within the Avon Longitudinal Study of Parents and Children (ALSPAC), which is described in detail elsewhere [30,31]. 916 of 1018 ARIES mother-offspring pairs had repeated methylation data which successfully passed quality control and of these 790 also had data on self-reported sustained smoking during pregnancy while of these again 744 also had complete covariate data. In unadjusted analysis conducted in [24], 15 CpG sites fell below the 1.07 × 10^−7^ Bonferroni threshold for significance and 28 CpG sites fell below the 0.05 false discovery rate cut-off. Where CpG sites fell below the Bonferroni threshold for significance and the association between sustained smoking and methylation was positive, we defined these sites as “hypermethylated”. Where CpG sites fell below the Bonferroni threshold for significance and the association between sustained smoking and methylation was inverse, we defined these sites as “hypomethylated”. We used multiple imputation to impute all missing data so that we could perform analyses on all 916 mother-child pairs. Since the parameter estimates from the BMM have a non-Gaussian distribution, they cannot be combined using Rubin’s rules. Hence we present results from one imputation, with four complementary sets of results given in online supplementary material. 

Whole-genome DNA methylation was determined using the Illumina Infinium^©^ HumanMethylation450 (HM450) BeadChip for methylation of over 485,000 key CpG sites [35]. In the original analyses by Richmond *et al.* unadjusted analyses identified 28 CpG sites that fell below the false discovery rate (FDR) cut-off of 0.05 [24] which were located in seven genes. 

### 2.2. Statistical Methods

The hierarchical BMM has previously been described in detail [32,33], but in summary describes a standard logistic model with all variables of interest (methylation of each CpG site (s)) and relevant confounders (γ_1_… γ_n_) included in the same model (hierarchical level 1 of the model) for individual *i*:

$$logit\left(p_{i}\right)=\alpha +\beta_{1}x_{i1}+\ldots +\beta_{S}x_{iS}+\gamma_{1}x_{i1}+\ldots +\gamma_{n}x_{in}$$

In addition to the assumption that maternal smoking during pregnancy has no effect on a proportion of CpG sites, we further assume a common prior for the effect sizes of the CpG sites that are affected by maternal smoking during pregnancy as well as prior distribution with its mass close to zero for those sites that are not affected by maternal smoking (level 2). 

Level 3 of the model describes the prior distribution of an indicator variable (T) that takes the value 1 if maternal smoking is associated with differential methylation at the CpG site and the value 0 if there is no association. 

This BMM describes a model in which maternal smoking during pregnancy has an effect on methylation in a proportion π of the measured CpG sites with the remaining proportion (*i.e.*, 1–π) of measured sites arising from a distribution with its mass close to zero (e.g., no effect). The proportion is included as the level 3 indicator value (T), and we define the Bayesian prior for T such that it has an independent Bernoulli distribution with the probability of “success” defined as the *a priori* hypothesized number of affected CpG sites (*i.e.*, π). In our example, we choose to fix this value because we have the results of previous analyses [24] which identified seven affected genes of 28 CpG sites (*i.e.*, we define prior probability π as 25%). 

We assume the effect of maternal smoking on methylation for each site arises from a mixture distribution that describes a relatively uninformative prior normal distribution N(0,σ_s_^2^) such that the Odds Ratio (OR; exp(β)) lies between 0.20 and 5 with probability 90% when T = 1, and, similarly, the parameter distribution N(0,σ_ns_^2^) for unaffected CpG sites (*i.e.*, T = 0) is set such that the Odds Ratio lies in the range 0.98–1.02 with 90% probability. The latter has been chosen, similar to previous work using this methodology, to account for residual confounding in the model. Specifically, level 2 can be described for each CpG site (s) as:

$$\beta_{s}|T_{s}^{S},\sigma_{S}^{2}\sim N\left(0,T_{s}^{S}\sigma_{S}^{2}+\left(1-T_{s}^{S}\right)\sigma_{ns}^{2}\right)$$

If we then average out over the mixture states, we obtain the mixture model results:

$$\beta_{s}|\pi_{s},\sigma_{S}^{2},\sigma_{ns}^{2}\sim \pi_{S}N\left(0,\sigma_{S}^{2}\right)+\left(1-\pi_{S}\right)N\left(0,\sigma_{ns}^{2}\right)$$

Each model is run for 50,000 samples after a 50,000 burn-in using Markov Chain Monte Carlo (MCMC) simulation. To reduce autocorrelation between the samples the MCMC chain was thinned by a factor 2. In these analyses we *a priori* assume that there is evidence of an association if the indicator variable shows that an association is present in more than 50% of the iterations of the MCMC chain, with an association present in a higher proportion of samples indicating stronger evidence of true association. Additionally, 95% credible intervals excluding unity are interpreted as stronger evidence for true association. Following recommendations in [32] multiple (n = 2) chains were run; one with starting values T = 0 and each regression parameter also set initially to zero and a second chain with all T’s set to 1, to enable assessment of mixing. Convergence was analyzed graphically based on trace, density and autocorrelation plots, as well as mathematically based on Brooks-Gelman-Rubin diagnostic statistics and MCMC errors. Additional sensitivity analyses were conducted specifying a prior probability π of 50% (which can be interpreted as an absence of prior information and thus a 50% probability for each CpG to be affected). 

The WinBUGS syntax for the BMM is provided in Online Supplementary Material.

The model also includes known (from [24]) confounding factors only. We further used independent surrogate variable analysis (ISVA) [36] to obtain the top 20 independent components of variation. These account for confounding due to position and/or batch effects, as well as changing cell type proportions which are to be expected when moving from cord blood to whole blood. The components have *a priori* been assigned an uninformative normal distribution. 

Similar to the analyses in Richmond *et al.* [24], also because the methodology has not yet been developed for longitudinal analyses, we conducted three cross-sectional analyses at birth, age 7 and age 17.

Note that in contrast to previous applications of the BMM in which any number of (correlated) environmental and/or genetic factors were assumed to result in increased odds of developing the disease of interest, in this context we take the approach that the dependent variable (Y) is maternal smoking, and that maternal smoking is associated with the set of “candidate” CpG sites; the independent variables (X). Thus we can fit one logistic model, and use this with the BMM approach to identify all the sites which are associated with smoking. From a causal perspective this is erroneous because of the directionality of the exposure-effect pathway—however, we are here primarily interested in associations rather than causation, to illustrate how the BMM might be used in epigenetic practice. Associations can be estimated even if the direction of causality is wrong—e.g., correlations (and their associated *p*-values) are unaffected by the direction of causality. This approach is analogous to that described in [37]. The alternative approach to the BMM would be to fit a linear regression model to each candidate CpG site, with maternal smoking as the exposure (as was done in [24]). 

## 3. Results

After imputation, the imputed ARIES dataset of the 916 pairs used in these analyses included 110 sustained smokers (Table 1). 110 sustained smokers during pregnancy (range in imputed sets 2–5 is 98–113) corresponds to 12%, which is comparable to the 11.5% in the non-imputed set [24] and similar to the population percentage reported previously for England [10]. Non-smoking mothers had, on average, higher education and higher social class than sustained smokers, were older on average, and were more likely to have smoking partners. Non-smoking mothers also drank alcohol more often on average, but had a higher percentage that stopped drinking after 18 weeks of gestation. Richmond *et al.* further describe [24] that, compared to the core ALSPAC sample, the ARIES offspring were more likely to be singletons, had a higher birth weight and had a longer gestation. The ARIES mothers were older at the time of delivery, had, on average, a higher education and were from a higher social class. They were also more likely to have drunk alcohol during pregnancy, but were less likely to have reported to have smoked in this period. 

Results of the methylation analyses using the BMM are shown in Table 2 for the first imputed dataset. The results for the remaining four imputed datasets are similar (online Supplementary Materials). Four of 28 CpG sites remain differentially methylated at birth in cord blood as a result of maternal smoking during pregnancy in 87%, 87%, 72% and 73% of samples, respectively (after adjustment for all other CpG sites and other confounding factors). One hypermethylated CpG site is located on the *MYO1G* gene (cg12803068) and three hypomethylated sites are located on the *GFI1* gene (cg09935388, cg06338710, cg09662411). There are further indications of differential methylation of the cg05575921 site on the *AHRR* gene, cg14179389 also on *GFI1* and the cg22549041 site located on the *CYP1A1* gene, but these signals are less strong and detected in 68%, 51% and 59% of MCMC samples, respectively. 

The differential methylation of cg12803068 on the *MYO1G* gene as a result of sustained maternal smoking during pregnancy remains consistent throughout childhood and adolescence, while the associations with methylation of *GFI1*, and to a lesser extent *CYP1A1* and *AHRR*, have either disappeared at age 7 (cg05575921, cg 14179389, cg063387100 and cg09662411) or slowly reversed (cg09935388) from birth until 7 years of age and further from 7 until the end of adolescence; both in effect size and in probability of observing an effect in the MCMC samples. Cg22132788 (*MYO1G*) is not differentially methylated at birth, but weak evidence of hypomethylation as a result of sustained maternal smoking is present at ages 7 and 17. Similarly, weak evidence of hypomethylation of cg18092474 (*CYP1A1*) is shown at age 17 only.

No associations between sustained smoking during pregnancy and DNA methylation were observed for *KLF13*, *ATP9A*, and *CNTNAP2*.

**Figure 1:**
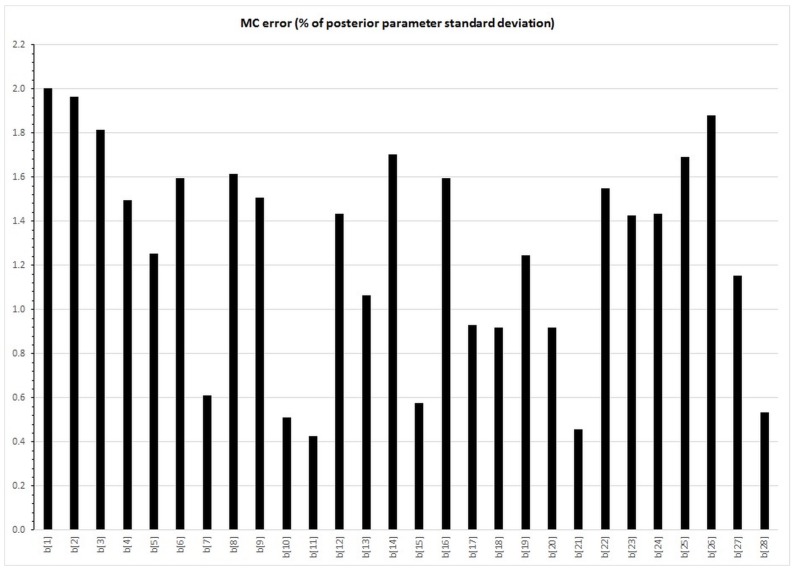
Monte Carlo (MC) errors for each CpG site’s model parameter (β_1_ to β_28_) as percentage of posterior parameter standard deviations; for cord blood imputed dataset 1 only.

**Figure 2:**
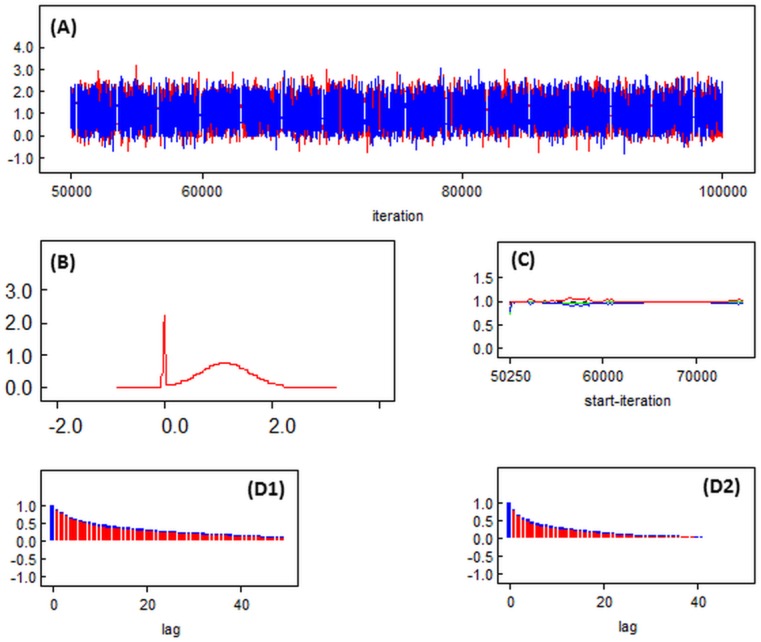
Diagnostic plots: trace plot (**A**); posterior density plot (**B**); Brooks-Gelman-Rubin (BGR) statistic plot (**C**); and autocorrelation plots in the absence of thinning (**D1**) and for thinning = 2 (**D2**)—for cord blood imputed dataset 1 CpG site cg12803068.

Each models took around 15 hours to run on a 64 bit system with a3.10 GHz processor and 8 Gb RAM. Diagnostics indicate good convergence with MC errors for all CpG site parameters <5% of parameter standard deviation (range 0.4%–2.0%) (Figure 1). Trace plots suggested good mixing, acceptable posterior densities clearly showing prior distributions for the null and non-null distributions. Brooks-Gelman-Rubin diagnostic statistics were stable near 1 indicating good convergence of the two mcmc chains. Autocorrelation existed, but was significantly reduced after thinning. Results were similar for the other imputed datasets. Diagnostic plots are shown graphically for the posterior cord blood results of CpG site (cg12803068) in Figure 2; shown for illustration and selected because it was the CpG site affected at all ages.

Sensitivity analysis indicated that prior choice of π (0.25 *vs.* 0.50) did not affect odds ratios or credible intervals, but did lead to increased probabilities for effect (Online Supplementary Material Table S1). As such, if, like in our analyses, the probability of effect (T) was used to signify effect, the choice of the prior for the Bernoulli distribution of T is important and should therefore be explicitly described. 

**Table 1:** Demographic characteristics of imputed ARIES dataset 1.

Characteristic	ARIES Dataset 1 (Missing Data Imputed)				ARIES Dataset (*)	
	Sustained Smoker		Non-Smokers		Sustained Smoker	Non-Smokers
	N	%	N	%	%	%
Sustained maternal smoking during pregnancy	110	12.0	804	87.9	11.5	88.5
Maternal education						
CSE/vocational	37	33.6	114	14.2	33.0	13.9
O-level	46	41.8	257	32.0	40.9	32.7
A-level	18	16.4	249	31.0	18.2	30.4
Degree	9	8.2	184	22.9	8.0	23.1
Maternal age						
<25 years	32	29.1	58	7.2	30.8	7.6
25–30	40	36.4	313	38.9	37.4	39.2
>30 years	38	34.5	433	53.9	31.9	53.2
Alcohol						
Non-drinker	43	39.1	269	33.5	40.9	34.2
Drank before 18 weeks gestation	5	4.5	122	15.2	5.7	15.6
Still drinking at 18 weeks of gestation	62	56.4	413	51.4	53.4	50.2
Paternal smoking	83	75.5	177	22.0	79.1	21.4
Household social class (manual labour)	38	34.5	72	9.1	35.4	8.8

* N varies according to completeness of data on baseline characteristics; see Richmond *et al* [24].

**Table 2:** Results of the hierarchical Bayesian mixture model in cord blood, at age 7 and age 17 years for imputed dataset 1.

CpG Site	Chromosome	Gene Region	Position	Cord blood			Age 7 Years			Age ~17 Years		
				Prob. of Effect (T) ^1^	OR ^2^	95% Cred. Limit ^3^	Prob. of Effect (T)	OR	95% Cred. Limit	Prob. of Effect (T)	OR	95% Cred. Limit (T)
cg05575921	5	*AHRR*	373 378	0.68	0.49	0.13–1.02	0.33	0.85	0.27–1.36	0.34	0.84	0.28–1.31
cg22132788	7	*MYO1G*	45 002 486	0.37	1.26	0.83–4.16	0.53	1.57	0.95–5.80	0.55	1.61	0.97–5.85
cg12803068	7	*MYO1G*	45 002 919	0.87	2.70	0.99–7.52	0.98	4.31	1.30–10.98	0.94	3.33	1.00–8.69
cg09935388	1	*GFI1*	92 947 588	0.87	0.36	0.12–1.01	0.57	0.65	0.22–1.02	0.43	0.76	0.27–1.05
cg14179389	1	*GFI1*	92 947 961	0.51	0.67	0.20–1.06	0.39	0.79	0.25–1.18	0.36	0.82	0.26–1.23
cg18146737	1	*GFI1*	92 946 700	0.38	0.80	0.26–1.14	0.24	0.99	0.48–1.84	0.24	0.99	0.50–1.82
cg05549655	15	*CYP1A1*	75 019 143	0.26	1.05	0.54–2.49	0.27	1.04	0.56–2.43	0.25	1.05	0.59–2.45
cg06338710	1	*GFI1*	92 946 187	0.72	0.54	0.20–1.02	0.20	1.02	0.68–1.75	0.22	0.95	0.49–1.29
cg12876356	1	*GFI1*	92 946 825	0.48	0.72	0.24–1.04	0.20	1.02	0.69–1.73	0.28	0.89	0.40–1.13
cg25949550	7	*CNTNAP2*	145 814 306	0.25	0.98	0.45–1.84	0.25	0.98	0.45–1.89	0.26	0.97	0.44–1.86
cg11902777	5	*AHRR*	3 68 843	0.25	0.99	0.46–1.92	0.26	0.98	0.46–1.95	0.25	0.99	0.47–1.94
cg12101586	15	*CYP1A1*	75 019 203	0.48	1.47	0.94–5.20	0.41	1.33	0.88–4.48	0.33	1.17	0.76–3.45
cg18316974	1	*GFI1*	92 947 035	0.22	0.98	0.51–1.68	0.25	0.96	0.42–1.72	0.24	0.97	0.45–1.69
cg26146569	15	*KLF13*	31 637 592	0.40	0.77	0.24–1.13	0.30	0.88	0.32–1.35	0.24	1.03	0.60–2.19
cg07339236	20	*ATP9A*	50 312 490	0.26	0.95	0.41–1.73	0.25	0.97	0.44–1.83	0.26	0.96	0.42–1.77
cg09662411	1	*GFI1*	92 946 132	0.73	0.48	0.15–1.02	0.20	1.01	0.64–1.67	0.21	1.04	0.73–1.93
cg18092474	15	*CYP1A1*	75 019 302	0.28	1.11	0.79–2.74	0.25	1.06	0.67–2.44	0.54	1.55	0.97–5.11
cg04180046	7	*MYO1G*	45 002 736	0.33	1.18	0.76–3.49	0.34	1.20	0.77–3.79	0.42	1.32	0.85–4.36
cg25189904	1	*GNG12*	68 299 493	0.41	0.76	0.23–1.16	0.34	0.85	0.28–1.32	0.24	0.99	0.49–1.87
cg04598670	7	*ENSG00000225718*	68 697 651	0.28	0.90	0.36–1.34	0.26	0.94	0.40–1.51	0.24	0.99	0.51–1.76
cg27629977	2	*CTNNA2*	80 531 633	0.25	1.03	0.56–2.29	0.26	1.01	0.50–2.11	0.25	0.99	0.47–1.97
cg10835306	9	*NOTCH1*	139 396 760	0.33	0.85	0.31–1.21	0.27	1.07	0.64–2.50	0.25	0.93	0.41–1.40
cg00483459	3	*ALS2CL*	46 735 782	0.34	0.85	0.28–1.29	0.24	1.01	0.53–2.07	0.27	1.08	0.63–2.70
cg22549041	15	*CYP1A1*	75 019 251	0.59	1.69	0.97–5.77	0.39	1.28	0.87–4.06	0.32	1.16	0.77–3.28
cg22937882	5	*AHRR*	4 05 774	0.29	1.12	0.71–3.06	0.26	0.95	0.40–1.65	0.25	0.99	0.48–1.89
cg11196333	1	*CHI3L1*	203 154 370	0.49	0.69	0.21–1.07	0.28	1.08	0.67–2.67	0.29	1.12	0.73–2.98
cg00624799	15	*ZNF710*	90 605 618	0.28	0.92	0.36–1.54	0.25	0.97	0.44–1.82	0.26	1.01	0.51–2.16
cg00560284	12	*SPATS2*	49 783 222	0.27	0.95	0.40–1.70	0.25	0.99	0.48–1.94	0.25	1.00	0.48–1.99

^**1**^ Probability of effect is the proportion of MCMC samples in which the BMM indicator value (T) indicated an association between sustained maternal smoking during pregnancy and differential methylation at the specific CpG site. Probability >50% was to determine association; ^**2**^ Odds Ratio; ^**3**^ 95% Credible Interval.

## 4. Discussion

We have shown that a Bayesian mixture model can be a useful statistical method to assess the effect of sustained maternal smoking during pregnancy on DNA methylation without the need of a further data reduction step to address correlated variables (after initial selection of 28 differentially methylated CpG sites); similar to its previously evaluated application for highly correlated data in the context of gene-environment interaction [32] and environmental exposure mixture studies [33]. 

We replicated the findings in Richmond *et al* [24] and similarly identified CpG sites in the *MYO1G* and *GFI1* genes to be hyper- and hypomethylated, respectively, as a result of sustained maternal smoking during pregnancy. These findings are also consistent with other previous publications indicating differential methylation between smokers and non-smokers [26,27,38]. This fits with biological evidence indicating that Myosin 1G (*MYO1G*) is involved in regulation of cell elasticity and associated with class 1 unconventional myosin expressed in hematopoietic cells, while growth factor independent 1 transcription repressor, encoded by the *GFI1* gene, plays a role in developmental disorders including hematopoiesis and oncogenesis. We further replicated, although the evidence from the BMM was less strong, effects on *CYP1A1* methylation as well as for hypomethylation of the cg05575921 site on the *AHRR* gene: *AHRR* mediates dioxin toxicity and this particular CpG site confirms previous findings also showing DNA methylation as a result of maternal smoking in cord and neonatal blood [16]. *CYP1A1* is involved in metabolism of polycylic aromatic hydrocarbons (PAHs) resulting from (e.g.,) tobacco smoking, and these are known to adversely influence lung cancer risk [39]. 

We did not replicate the hits located on the *KL13*, *ATP9A*, and *CNTNAP2* genes reported by Richmond *et al.* [24]. Recent data similarly did not show differential methylation of CpG sites on *KL13* and *ATP9A* in Dutch children, but contrary to our findings did identify *CNTNAP2* [38], while contrary to the BMM results *ATP9A* was also identified as a smoking responsive locus in a recent study in Norway [17].

Although the results of these analyses and those reported by Richmond *et al.* [24] largely overlap there are some differences, for which there are two likely explanations: either the hits that were not replicated using the BMM may have been false positive findings in the original analyses as a result of CpG site selection and correlations between sites, or the BMM results include several type II errors as a result of model specification or insufficient statistical power. The former is supported by results of a study in which BMM performance was evaluated using simulated datasets with comparable variable distributions and correlations to those of the epigenetic data analysed here, and which indicated that the occurrence of type I errors was minimal [33]. At the same time, the simulations showed that type II errors in the BMM cannot be excluded based on these analyses alone; replication in other studies in this respect will be important. 

Cellular heterogeneity between cord blood and whole blood is one limitation of the current analysis. Cell type proportions are different in these two tissue types and this change over time could account for changes in methylation observed over the same period. In ARIES the cell type proportions were not measured, so we were unable to directly account for this possibility. However, we used independent surrogate variable analysis to obtain components of variation which are likely to account for the changes in cell type proportions over time. 

Another limitation of these data is tissue specificity because, in epigenetic research, levels of methylation vary between tissue types. In the current study we have blood sample methylation but it may be have been more informative had we been able to test the effect of smoking on methylation using other tissue types such as, for example, saliva or bronchial epithelium [40]. 

Although we have demonstrated the benefits of using a BMM to analyse moderately to highly correlated epigenetic data, an important limitation of the use of this methodology is that, in contrast to conventional, frequentist methods, obtaining the results from 100,000 MCMC iterations can take quite a long time. A further issue is that the use of the mixture model results in skewed, non-Gaussian, model parameters and as a result Rubin’s rules for combination of the results of the five imputed datasets do not apply (this can also be incorporated in the model, but for the purpose of these analyses we chose to present the results individually for each set) [41]. Thirdly, it has been shown that the ratios of the variances for the priors should not be too large since this may result in poor convergence of the Markov chain as a result of the sampler getting “stuck” at one of the T states [42]. As indicated by our model diagnostics our *a priori* choice for relatively uninformative prior for non-null effects (OR range 0.2–5) compared to that for null effects (OR range 0.98–1.02) resulted in good convergence, but in those situations where this is not the case more informative priors with smaller variance may be a solution. 

And finally, in these analyses we only included the 28 CpG sites with differential methylation. Preferably, no *a priori* variable selection should be conducted, but the performance of this BMM has not yet been evaluated beyond several hundreds of included variables [32,33] falling short of the over 485,000 key CpG sites on the Illumina Infinium^©^ HumanMethylation450 (HM450) BeadChip.

We could have extended the BMM to include methylation from the CpG sites at all the three timepoints in once model, rather than three separate models. However, this might have been complicated by the fact that the priors for the same CpG site would need to be allowed to be correlated. Alternative statistical methods to the BMM could have been used to model the longitudinal nature of these data, such as for example generalized linear mixed models (GLMM) or generalized estimation equations (GEE). The GLMM and GEE models can model DNA methylation across three time points, allowing changes in DNA methylation with time to be estimated. However, since there are 28 CpG sites, this would require 28 different models, leading to issues of power and false positive rates. 

The ARIES data are unique in having three serial measures of DNA methylation across childhood and adolescence on 1018 children. Together with a plethora of information on both mothers and children contained in the ALSPAC cohort, the data used for analysis are a major strength of this article. Future work could include the application of the BMM approach to other exposures or sources of DNA methylation variation within this data series as well as in other cohort studies.

Further improvements of the BMM would lie in the addition of another hierarchical level that allows for correlation of methylation of CpG sites within CpG islands [43] or located within the same genes, as well as further expansion of this framework for longitudinal analyses. In these analyses we used three cross-sectional models to compare methylation patterns at birth, age 7 and in adolescence, but with better characterization of the dynamic elements of the human methylome [44], longitudinal analyses will help to better elucidate persistent and reversible effects of (environmental) exposures as well as critical periods of effect [45]. 

This study demonstrated that the use of the BMM can be a useful addition to the statistical methodologies to analyse epigenetic datasets. Here we showed that the identification of specific differentially methylated CpG sites related to sustained maternal smoking during pregnancy was comparable to those identified in Richmond *et al.* [24], but did not require a second pre-analysis data reduction step in which potentially important sites could be missed because they were not included. Moreover, the mixture model applied by Richmond *et al.* indicated some possibly false positive findings, most notably those for the *KL13* and *ATP9A* genes, which is in line with findings elsewhere [38]. However, although the BMM greatly reduced the occurrence of type I errors, because type II errors cannot be excluded we recommend using this methodology in addition to other statistical methods rather than using it as a replacement. 

## 5. Conclusions

These analyses demonstrated the benefits of using the BMM for analyses of CpG sites deemed to be epigenome-wide significant in downstream analyses in a Bayesian context rather than (arbitrarily) focusing on the top CpG sites in each gene region which may miss other interesting CpG sites, and as such this method may be useful for prioritising CpG sites in other large-scale EWAS studies.

## Supplementary Files

Supplementary File 1
