# Supplementary material for: Assessment of Offspring DNA Methylation across the Lifecourse Associated with Prenatal Maternal Smoking Using Bayesian Mixture Modelling
Source: Int J Environ Res Public Health. 2015 Nov 13;12(11):14461–76. doi: 10.3390/ijerph121114461 (PMC4661660; doi:10.3390/ijerph121114461)
Supplement: Supplementary File 1 [file ijerph-12-14461-s001.pdf]

Supplementary Information

Assessment of Offspring DNA Methylation across the Lifecourse Associated with Prenatal Maternal Smoking Using Bayesian Mixture Modelling

---

Hierarchical Bayesian Mixture Model WinBUGS syntax

```
model {
for (i in 1 : 914) {
y[i]~dbern(p[i])

# Logistic linear predictor
logit(p[i]) <- alpha + s1[i]+s2[i] +s3[i]+e1*conf14[i]+e2* conf13[i]+e3*
conf12[i]+e4*conf22[i]+e5*conf21[i]+e6*conf32[i]+e7*
conf31[i]+e8*conf4[i]+e9*conf5[i]+d1[i]+d2[i]

s1[i]<-b[1]*cg05575921[i]+
b[2]*cg22132788[i]+
b[3]*cg12803068[i]+
b[4]*cg09935388[i]+
b[5]*cg14179389[i]+
b[6]*cg18146737[i]+
b[7]*cg05549655[i]+
b[8]*cg06338710[i]+
b[9]*cg12876356[i]+
b[10]*cg25949550[i]
s2[i]<- b[11]*cg11902777[i]+
b[12]*cg12101586[i]+
b[13]*cg18316974[i]+
b[14]*cg26146569[i]+
b[15]*cg07339236[i]+
b[16]*cg09662411[i]+
b[17]*cg18092474[i]+
b[18]*cg04180046[i]+
b[19]*cg25189904[i]+
b[20]*cg04598670[i]
s3[i]<-b[21]*cg27629977[i]+
b[22]*cg10835306[i]+
b[23]*cg00483459[i]+
b[24]*cg22549041[i]+
b[25]*cg22937882[i]+
```

```
b[26]*cg11196333[i]+
b[27]*cg00624799[i]+
b[28]*cg00560284[i]

# independent surrogate variables
d1[i]<-
g[1]*isv1[i]+g[2]*isv2[i]+g[3]*isv3[i]+g[4]*isv4[i]+g[5]*isv5[i]+g[6]*isv6[i]+g[7]*isv7[i]+g[8]*isv8
[i]+g[9]*isv9[i]+g[10]*isv10[i]
d2[i]<-
g[11]*isv11[i]+g[12]*isv12[i]+g[13]*isv13[i]+g[14]*isv14[i]+g[15]*isv15[i]+g[16]*isv16[i]+g[17]*i
sv17[i]+g[18]*isv18[i]+g[19]*isv19[i]+g[20]*isv20[i]
}

# Improper flat prior on the intercept
alpha~dflat()
# uninformative priors confounders
e1~dnorm(0,000.1)
e2~dnorm(0,000.1)
e3~dnorm(0,000.1)
e4~dnorm(0,000.1)
e5~dnorm(0,000.1)
e6~dnorm(0,000.1)
e7~dnorm(0,000.1)
e8~dnorm(0,000.1)
e9~dnorm(0,000.1)
# Priors for the CpG effects
for (j in 1:28){
prec[j]<- 1/(T[j]*0.310+(1-T[j])*0.000145)
b[j]~dnorm(0,prec[j])
T[j]~dbern(0.25)
}
for (k in 1:20){
g[k]~dnorm(0,0.0001)
}
}
#initial starting point 1
#initial starting point 2
#DATA
```

Table S1. Sensitivity analysis BMM for cord blood data using a prior Bernoulli distribution with probability (π) of success of 25% for indicator value *T* (original analyses; Table 2) compared to π = 50%.

				T ~ Bernoulli(0.25)			T ~ Bernoulli(0.50)		
**CpG site**	**Chromosome**	**Gene region**	**Position**	**Prob. of effect (T) [1]**	**OR [2]**	**95% Cred. Limit [3]**	**Prob. of effect (T) [1]**	**OR [2]**	**95% Cred. Limit [3]**
cg05575921	5	*AHRR*	373 378	0.68	0.49	0.13–1.02	0.84	0.43	0.13–1.03
cg22132788	7	*MYO1G*	45 002 486	0.37	1.26	0.83–4.16	0.63	1.45	0.71–4.59
cg12803068	7	*MYO1G*	45 002 919	0.87	2.70	0.99–7.52	0.93	2.75	0.99–7.30
cg09935388	1	*GFI1*	92 947 588	0.87	0.36	0.12–1.01	0.92	0.37	0.13–1.01
cg14179389	1	*GFI1*	92 947 961	0.51	0.67	0.20–1.06	0.74	0.57	0.18–1.20
cg18146737	1	*GFI1*	92 946 700	0.38	0.8	0.26–1.14	0.59	0.74	0.26–1.43
cg05549655	15	*CYP1A1*	75 019 143	0.26	1.05	0.54–2.49	0.51	1.09	0.47–2.97
cg06338710	1	*GFI1*	92 946 187	0.72	0.54	0.20–1.02	0.82	0.53	0.21–1.02
cg12876356	1	*GFI1*	92 946 825	0.48	0.72	0.24–1.04	0.67	0.66	0.24–1.20
cg25949550	7	*CNTNAP2*	145 814 306	0.25	0.98	0.45–1.84	0.50	0.96	0.37–2.27
cg11902777	5	*AHRR*	3 68 843	0.25	0.99	0.46–1.92	0.50	0.97	0.38–2.38
cg12101586	15	*CYP1A1*	75 019 203	0.48	1.47	0.94–5.20	0.72	1.72	0.81–5.45
cg18316974	1	*GFI1*	92 947 035	0.22	0.98	0.51–1.68	0.45	1.00	0.46–2.19
cg26146569	15	*KLF13*	31 637 592	0.40	0.77	0.24–1.13	0.65	0.67	0.22–1.34
cg07339236	20	*ATP9A*	50 312 490	0.26	0.95	0.41–1.73	0.52	0.91	0.33–2.09
cg09662411	1	*GFI1*	92 946 132	0.73	0.48	0.15–1.02	0.84	0.46	0.16–1.02
cg18092474	15	*CYP1A1*	75 019 302	0.28	1.11	0.79–2.74	0.51	1.18	0.64–3.00
cg04180046	7	*MYO1G*	45 002 736	0.33	1.18	0.76–3.49	0.58	1.31	0.64–3.97
cg25189904	1	*GNG12*	68 299 493	0.41	0.76	0.23–1.16	0.66	0.66	0.21–1.34
cg04598670	7	*ENSG00000225718*	68 697 651	0.28	0.9	0.36–1.34	0.53	0.84	0.31–1.65
cg27629977	2	*CTNNA2*	80 531 633	0.25	1.03	0.56–2.29	0.51	1.06	0.45–2.81
cg10835306	9	*NOTCH1*	139 396 760	0.33	0.85	0.31–1.21	0.57	0.78	0.29–1.44
cg00483459	3	*ALS2CL*	46 735 782	0.34	0.85	0.28–1.29	0.58	0.76	0.25–1.53
cg22549041	15	*CYP1A1*	75 019 251	0.59	1.69	0.97–5.77	0.80	1.96	0.91–5.93
cg22937882	5	*AHRR*	4 05 774	0.29	1.12	0.71–3.06	0.56	1.23	0.57–3.65
cg11196333	1	*CHI3L1*	203 154 370	0.49	0.69	0.21–1.07	0.72	0.59	0.20–1.18
cg00624799	15	*ZNF710*	90 605 618	0.28	0.92	0.36–1.54	0.53	0.86	0.30–1.86
cg00560284	12	*SPATS2*	49 783 222	0.27	0.95	0.40–1.70	0.52	0.91	0.33–2.06

[1] Probability of effect is the proportion of MCMC samples in which the BMM indicator value (T) indicated an association between sustained maternal smoking during pregnancy and differential methylation at the specific CpG site; [2] Odds Ratio; [3] 95% Credible Interval.

© 2015 by the authors; licensee MDPI, Basel, Switzerland. This article is an open access article distributed under the terms and conditions of the Creative Commons Attribution license (http://creativecommons.org/licenses/by/4.0/).
